# Improving Reconstituted HDL Composition for Efficient Post-Ischemic Reduction of Ischemia Reperfusion Injury

**DOI:** 10.1371/journal.pone.0119664

**Published:** 2015-03-17

**Authors:** Marie-Claude Brulhart-Meynet, Vincent Braunersreuther, Jonas Brinck, Fabrizio Montecucco, Jean-Christophe Prost, Aurelien Thomas, Katia Galan, Graziano Pelli, Sarah Pedretti, Nicolas Vuilleumier, François Mach, Sandrine Lecour, Richard W. James, Miguel A. Frias

**Affiliations:** 1 Service of Endocrinology, Diabetology, Hypertension and Nutrition, Department of Internal Medicine, Faculty of Medicine, Geneva, Switzerland; 2 Division of Cardiology, Department of Internal Medicine, Geneva University Hospital, Geneva, Switzerland; 3 Service of Laboratory Medicine, Department of Genetics and Laboratory Medicine, Geneva University Hospital, Geneva, Switzerland; 4 Department of human protein sciences, Geneva University Hospital, Geneva, Switzerland; 5 First Clinic of Internal Medicine, Department of Internal Medicine, University of Genoa School of Medicine. IRCCS Azienda Ospedaliera Universitaria San Martino—IST Instituto Nazionale per la Ricerca sul Cancro, Genoa, Italy; 6 Unit of Toxicology, CURML, University of Lausanne, Lausanne, Switzerland; 7 Hatter Institute for Cardiovascular Research in Africa, Faculty of Health Science, University of Cape Town, Cape Town, South Africa; University of Milano, ITALY

## Abstract

**Background:**

New evidence shows that high density lipoproteins (HDL) have protective effects beyond their role in reverse cholesterol transport. Reconstituted HDL (rHDL) offer an attractive means of clinically exploiting these novel effects including cardioprotection against ischemia reperfusion injury (IRI). However, basic rHDL composition is limited to apolipoprotein AI (apoAI) and phospholipids; addition of bioactive compound may enhance its beneficial effects.

**Objective:**

The aim of this study was to investigate the role of rHDL in post-ischemic model, and to analyze the potential impact of sphingosine-1-phosphate (S1P) in rHDL formulations.

**Methods and Results:**

The impact of HDL on IRI was investigated using complementary *in vivo*, *ex vivo* and *in vitro* IRI models. Acute post-ischemic treatment with native HDL significantly reduced infarct size and cell death in the *ex vivo*, isolated heart (Langendorff) model and the *in vivo* model (-48%, p<0.01). Treatment with rHDL of basic formulation (apoAI + phospholipids) had a non-significant impact on cell death *in vitro* and on the infarct size *ex vivo* and *in vivo*. In contrast, rHDL containing S1P had a highly significant, protective influence *ex vivo*, and *in vivo* (-50%, p<0.01). This impact was comparable with the effects observed with native HDL. Pro-survival signaling proteins, Akt, STAT3 and ERK1/2 were similarly activated by HDL and rHDL containing S1P both *in vitro* (isolated cardiomyocytes) and *in vivo*.

**Conclusion:**

HDL afford protection against IRI in a clinically relevant model (post-ischemia). rHDL is significantly protective if supplemented with S1P. The protective impact of HDL appears to target directly the cardiomyocyte.

## Introduction

Ischemia reperfusion injury (IRI) is a major concern for cardiologists in the treatment of myocardial infarction. Limiting the extent of IRI is of primary importance in preventing subsequent progression to heart failure. The timing of clinical intervention is crucial and for obvious reason, preconditioning is not feasible. It is therefore important to evaluate the application of efficient treatment at the adequate moment.

For several decades, high density lipoproteins (HDL) have been considered a strong negative predictor of cardiovascular risk, with an inverse relationship to atherosclerotic disease risk. This beneficial effect on the cardiovascular system has been attributed primarily to its ability to facilitate cholesterol excretion [1]. Recent evidence has shown that HDL particles have more widespread, beneficial actions (antioxidant, anti-inflammatory, anti-apoptotic) on vascular cells [1]. Direct actions of HDL on the heart have been poorly investigated although experimental data have shown that HDL can influence IRI [2, 3]. These effects have been attributed to several components of the HDL particle, which is complex and heterogeneous (more than 50 peptides and 100 lipids have been shown to be associated with HDL). Although apolipoprotein AI (apoAI) constitutes more than 70% of the total protein content in HDL particles, it appears too simplistic to attribute to apoAI all the beneficial effects.

The HDL complex is being increasingly considered a therapeutic option, notably in the form of synthetic (reconstituted) HDL (rHDL) whose composition can be controlled and which, in basic formulations, contains apoAI and phospholipids. It has already been investigated in several experimental settings in man, although not against IRI.

In the experimental animal model of IRI, administration of rHDL before ischemia improves post-ischemic cardiac recovery, while the protective impact was reportedly limited when administrated at reperfusion [2, 4, 5]. The rHDL used in these studies only contained apoAI and phospholipids.

Sphingosine-1-phosphate (S1P) is a lipid active on vascular cells. It has been shown to confer protection against IRI. Interestingly, S1P is a constituent of the HDL particle and the level of S1P in HDL particle is decreased in patients with coronary artery disease [6, 7]. Studies of the role of HDL and S1P in protection against IRI *in vivo* are limited to one report, where HDL were administered prior to ischemia [3]. It employed native HDL (ie isolated from human plasma) and S1P infused independently of HDL. We hypothesised that the artificial addition of S1P to the basic rHDL formulation could potentiate its action and improve their therapeutic impact.

The aim of the present study was to extend our investigations of HDL to more physiologically relevant models of IRI to (i) investigate its protective effect in a post-ischemic, *in vivo* model, (ii) explore the impact of rHDL and determine whether addition of S1P to the basic rHDL formulation could improve cardioprotection, (iii) investigate molecular mechanisms, comparing the simplified *in vitro* with more physiologically relevant *ex vivo* and *in vivo* models.

Our results underline the efficacy of HDL protection of the heart in a post-ischemic context, which is the most appropriate timing for clinical intervention against acute coronary disease. They demonstrate that a minimal composition of rHDL requires S1P to achieve cardioprotection equivalent to that of native HDL. Our data suggest that the protective effect is primarily mediated by a direct impact on cardiomyocytes. Finally, we demonstrate activation of similar, protective signaling pathways in the *in vitro* and *in vivo* models of IRI.

## Materials and Methods

### Animals

All experiments involving animals were approved by the Animal Research Ethics Committee from the faculty of health sciences of University of Cape Town (UCT) for experiments done at UCT and by the animal ethical committee of the Geneva University Medical School for experiments done in Geneva. Animals were housed and treated in accordance with the Guide for Care and Use of laboratory Animals Eighth Edition, published by the US National Institute of Health Publication. Male C57black6 mice aged 8–14 weeks and neonatal Wistar rats were used in this study.

### HDL isolation

HDL (d = 1.063–1.21 g/mL) were isolated by cumulative flotation ultracentrifugation as previously described [8] from a plasma pool provided by healthy volunteers (authorized by the ethics committee of the University Hospital, Geneva, authorization n° 07–037), dialyzed against phosphate buffered saline (PBS) containing ethylenediaminetetraacetic acid (EDTA) (0.1mM) and stored at 4°C. The volunteers provided informed, written consent in accordance with the PLOS ONE editorial policy.

HDL particles were also isolated from serum mouse. In short, apoB-containing lipoproteins were removed from 200μL serum by precipitation with 4g/L Na-phosphotungstate and 50mmol/L MgCl_2_ (final concentration after being added to the sample). HDL particles were isolated by ultracentrifugation (TLA-100 rotor, 100′000rpm, 5h, 4°C, Beckman Coulter centrifuge). HDL protein concentration was measured by Lowry.

### rHDL preparation

ApoAI was isolated from delipidated human HDL [8]. Palmitoyloleophosphatidyl choline (POPC) was purchased from Sigma-Aldrich GmbH (Buchs, Switzerland) and Santa Cruz (Heidelberg, Germany). Two different forms of rHDL were prepared by the cholate dialysis procedure [9], maintaining a POPC/apoAI molar ratio of 100:1: rHDL containing POPC and apoAI and rHDL with addition of S1P (rHDLB) with a POPC/apoAI/S1P molar ration of 1600/16/1 (Sigma-Aldrich GmbH (Buchs, Switzerland). For preparations containing S1P, aliquots from a stock solution (1.0 mg/mL in methanol) were dried in glass tubes and the POPC/cholate mixture, and then apoAI were added successively, as prescribed by the cholate dialysis procedure. All preparations were dialyzed 4 days against Tris 10 mM, pH 8.0, NaCl, 0.15M. The S1P concentration in rHDLB was 74.8ng/mg apoAI, slightly higher than S1P contained in native HDL (59ng/mg apoAI) (see S1 Fig.). The amount of S1P injected corresponds to an estimated final concentration of 1.2μM.

### S1P measurement in HDL

HDL content of S1P was analyzed by liquid-chromatography/tandem mass spectrometry (LC/MS—MS) (see S1 Methods for details).

### Cell culture

Isolation of neonatal ventricular cardiomyocytes was performed with 1 to 2-day-old Wistar rats. Cells were isolated by digestion with trypsin-EDTA and collagenase as described previously in detail [10].

### Hypoxia protocol

Before hypoxic exposure, cell medium was replaced by modified Tyrode’s solution (in mM: NaCl 136.9, KCl 2.68, Na_2_HPO_4_^.^12H_2_O 8.1, KH_2_PO_4_ 1.47, CaCl_2_ 0.9, MgCl_2_^.^6H_2_O 0.49; pH 7.2). The cardiomyocytes were placed in an anaerobic hypoxia chamber containing 5% CO_2_ and 95% N_2_ at 37°C for 5h. For normoxic treatment cells were maintained at 37°C in a culture incubator with 5% CO_2_. After treatment, cell survival was evaluated by the colorimetric MTT assay (3-[4, 5-dimethylthiazol-2-yl]-2,5-diphenyl tetrazolium bromide; Calbiochem; La Jolla, California, USA).

### Perfusion of isolated mouse hearts (Fig. 1A)

**Fig 1:**
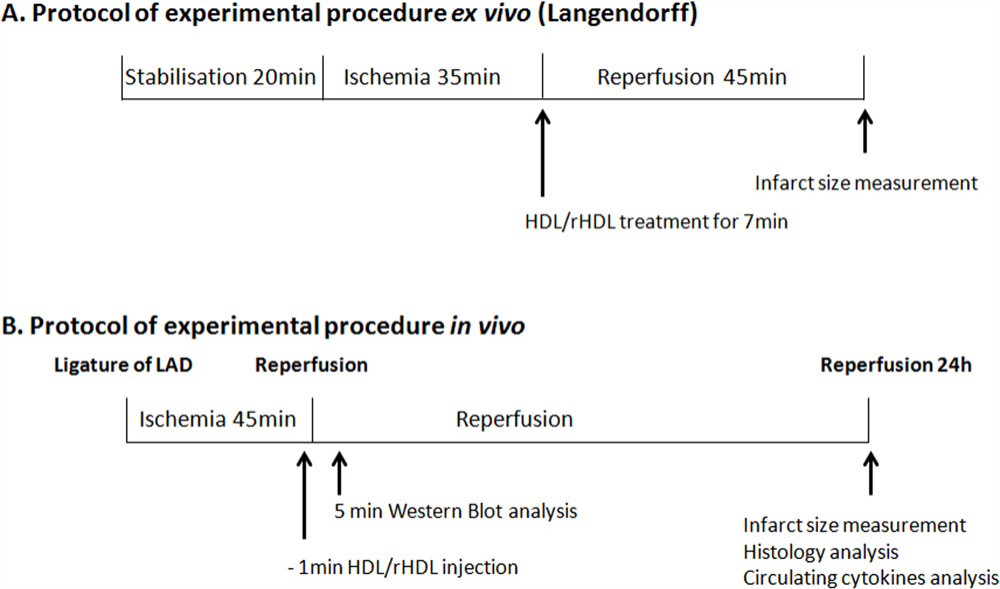
Experimental protocols. **A.** Hearts from mice were submitted to the ischemia reperfusion protocol as follows: 20min of stabilization, 35min of no-flow global ischemia and 45min of reperfusion. After reperfusion, infarct size was determined. Native HDL or rHDL were added for the first 7min of reperfusion. **B.** Mice were subjected to LAD occlusion for 45min and hearts were reperfused for different time periods: 5min for Western blot experiments and 24h for infarct size measurement and histological analysis. Serum from mice reperfused for 24h was collected for analysis of circulating cytokines. Native HDL or rHDL were injected one min before reperfusion.

Mice were anaesthetized via an intraperitoneal injection of sodium pentobarbital (60mg/kg). The heart was quickly excised and the aorta cannulated for retrograde perfusion in a Langendorff apparatus. Isolated hearts were subjected to a stabilization period (20min) followed by global ischemia (35min) then a reperfusion period (45min) as previously described [11]. At the onset of reperfusion, HDL were perfused for 7min via a side pump adjusted to deliver a specified concentration of HDL (native HDL, 200μg protein/ml; rHDL, apoAI at 140μg/ml, S1P at 500nM). At the end of reperfusion, the hearts were sliced and stained with triphenyltetrazolium chloride (TTC) 1%. Infarct size (IS) was evaluated by the analysis of the dead and living cell areas normalized to the total heart area using computerized planimetry (Planimetry+, Boreal Software, Norway). A minimum of 4 hearts was used in all groups.

### *In vivo* ischemia reperfusion protocol (Fig. 1B)

*In vivo* IRI was analyzed after ligation of the left anterior descending (LAD) coronary artery. In brief, mice were anaesthetized with 4% isoflurane and intubated. Mechanical ventilation was performed (150μl, 120 breaths/min) using a rodent respirator (model 683; Harvard Apparatus). Anaesthesia was maintained with 2% isoflurane delivered in 100% O_2_ through the ventilator. A thoracotomy was performed and an 8–0 prolene suture was passed under the left anterior descending (LAD) coronary artery. After 45min ischemia, LAD coronary artery occlusion was released and reperfused for different times. After 24h of reperfusion the mice were re-anaesthetized with ketamine—xylazine (120mg/kg and 16mg/kg, respectively) and the LAD coronary artery was re-occluded. Evan’s blue dye 2% (Sigma) was injected to delineate the *in vivo* area at risk (AAR). The heart were sliced into 5–6 sections and stained with TTC 1% to allow quantification of infarct size. The different zones were determined using a computerized planimetric technique (MetaMorph v6.0, Universal Imaging Corporation) and infarct size was expressed in percentage of AAR (I/AAR). HDL were injected intravenously (retro-orbital) one minute before reperfusion. For injection, native HDL was used at 100μg protein/g mouse; for rHDL, apoAI was 80μg/g mouse and S1P was 57ng/g mouse. 80μg of apoAI approximates the amount of apoAI present in 100μg protein of native HDL.

### Serum cytokine measurement

Sera from PBS, native HDL or rHDLB (rHDL+ S1P) treated mice were collected after 24h reperfusion to assess cytokine concentrations. CCL2 and CXCL1 were measured using ELISA procedures (R&D Systems, Abingdon, UK), according to the manufacturer’s guidelines. The limit of detection was 15.6pg/ml (CXCL1) and 7.8pg/ml (CCL2). Mean intra- and inter-assay coefficients of variation (CV) were below 6%.

### Histological analysis

Hearts isolated from animals were perfused with NaCl 0.9% to remove blood and frozen in OCT. They were then cut serially from the occlusion locus to the apex in 7μm sections. In order to obtain an appropriate and complete sampling of ischemic cardiac regions, five frozen midventricular cardiac sections (between the coronary ligature and the apex) per animal (euthanized after 24h of reperfusion) distant 630μm from each other were stained and analyzed.

### Inflammatory cell recruitment

Mouse heart sections obtained as described above were stained for neutrophils with rat anti-mouse neutrophil Ly-6B.2 (MCA771G; Serotec) or with anti-mouse neutrophil Ly6G+ (# 551459, BD Pharmingen) antibodies. CD68^+^ macrophages were stained with rat anti-mouse CD68 (MCA1957GA; Serotec) antibody. For each heart, the number of cells was counted using MetaMorph v6.0 (Universal Imaging Corporation) on 5 midventricular cardiac sections per animal, as previously described [12, 13]. Results are expressed as percentages of stained area on total heart surface area.

### Myocardial oxidative stress determination (24h after reperfusion)

Neutrophil, monocyte and eosinophil activation is known to catalyse the formation of hypochlorous acid that reacts with proteins and induces tyrosine halogenation such as 3,5-dibromotyrosine (Di-BrY). Leukocyte-derived oxidative stress was assessed after 24h of reperfusion in frozen sections of the ischemic hearts by immunostaining for 3,5-dibromotyrosine using a mouse anti-dibromotyrosine monoclonal antibody (at 10μg/ml, AMS biotechnology, LTD, Abingdon, UK). To avoid any potential cross-reactivity with mouse heart antigens and to increase the specificity of the primary antibodies, the VECTOR M.O.M immunodetection kit and the VECTOR VIP substrate kit for peroxidase (Vector Laboratories, Inc. Burlingame, CA) were used, following the manufacturer’s instructions.

### Western blot analysis

For protein analysis of *in vivo* treated mice, hearts were removed after 5min reperfusion, snap-frozen in liquid nitrogen then stored at −80°C until protein extraction was performed.

Total proteins were extracted from the whole heart by homogenization of the tissue in lysis buffer containing Tris-HCl (50mM, pH 7.4), NaCl (150mM), glycerol (10%v/v), EDTA (2mM), EGTA (2mM), Triton X-100 (1%v/v), b-glycerophosphate (40mM), NaF (50mM) and a mixture of protease inhibitors (Roche, Mannheim, Germany) and phenyl-methyl-sulfonyl fluoride (1mM). Cardiomyocytes from *in vitro* studies were treated 5min with HDL (200μg/ml) or rHDL (apoAI: 160μg/ml). At the end of the treatment cells were washed with PBS and lysed with lysis buffer. Phosphorylated Akt (Phospho-Akt, serine 473, rabbit polyclonal, cat n° 9271, Cell Signaling), extracellular regulated-signal kinase 1/2 (ERK1/2) (Phospho-ERK1/2, rabbit polyclonal, cat n° 9101, Cell Signaling), Signal Transducer and Activator of Transcription 3 (STAT3) (Phospho-STAT-3 serine727: STAT3-S727 cat n° 9134 and phospho-STAT3 tyrosine 705: STAT3-Y705, cat n° 9131, both rabbit polyclonal from Cell Signaling), were analyzed by sodium dodecyl sulfate-polyacrylamide gel electrophoresis (SDS—PAGE). Levels of phosphorylated proteins were normalized to glyceraldehyde 3-phosphate dehydrogenase (GAPDH, mouse monoclonal antibody cat n° MAB374 from Millipore, Billerica, MA, USA). For respective total kinase protein expression the membranes were stripped and reprobed with total STAT3 anti-rabbit, n° cat 06–596 Millipore), total ERK (rabbit polyclonal, n° cat sc-94, Santa Cruz Biotechnology, Dallas, TX, USA) and total Akt (rabbit polyclonal, cat n° 9272, Cell Signaling). Relative densities were determined and quantified with the Odyssey Imager (Li-Cor Biosciences).

### Statistical analysis

All statistical analyses were performed using the software GraphPad Prism 6. Data are expressed as mean ± the standard error of the mean (SEM). Comparisons were performed by unpaired or paired Student t-test (two tail) as well as one-way ANOVA combined with Tukey’s multiple comparisons post-hoc test where appropriate. p<0.05 was considered significantly different.

## Results

### rHDL containing S1P induce cardioprotection in a hypoxia model

**Fig 2:**
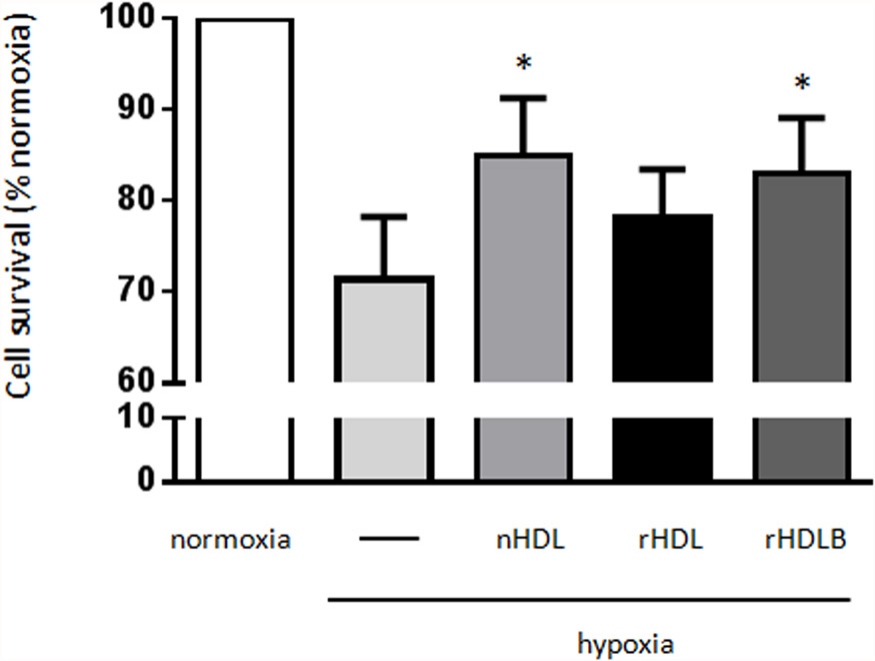
rHDL containing S1P protect against hypoxia *in vitro*. Cardiomyocytes were incubated in Tyrode solution, submitted to 5h hypoxia and treated during hypoxia with native HDL (nHDL), rHDL (apoAI+POPC) or rHDLB (apoAI + POPC + S1P). Cell survival was determined using the MTT assay. Cell survival is expressed in percentage of normoxia (mean±SEM). *p < 0.05 vs hypoxia, using paired-student t-test, n = 6.

Our previous *in vitro* studies on the protective effects of HDL employed rat cardiomyocytes subjected to pathophysiological insult by doxorubicin. To furnish *in vitro* data on ischemia more relevant to our *ex vivo* and *in vivo* models, we re-examined the effects of native HDL and rHDL on cardiomyocytes under conditions of hypoxia. As shown in Fig. 2, hypoxia induced a significant level of cell death (30%, p<0.05). While basic rHDL (POPC+apoAI) did not significantly improve cell survival after hypoxia, native HDL and addition of S1P to the basic rHDL formulation (rHDL+S1P = rHDLB) reduced cell death by 47%±11 and 41% ±19, respectively (p<0.05).

### rHDL containing S1P induce cardioprotection *ex vivo* and *in vivo*

**Fig 3:**
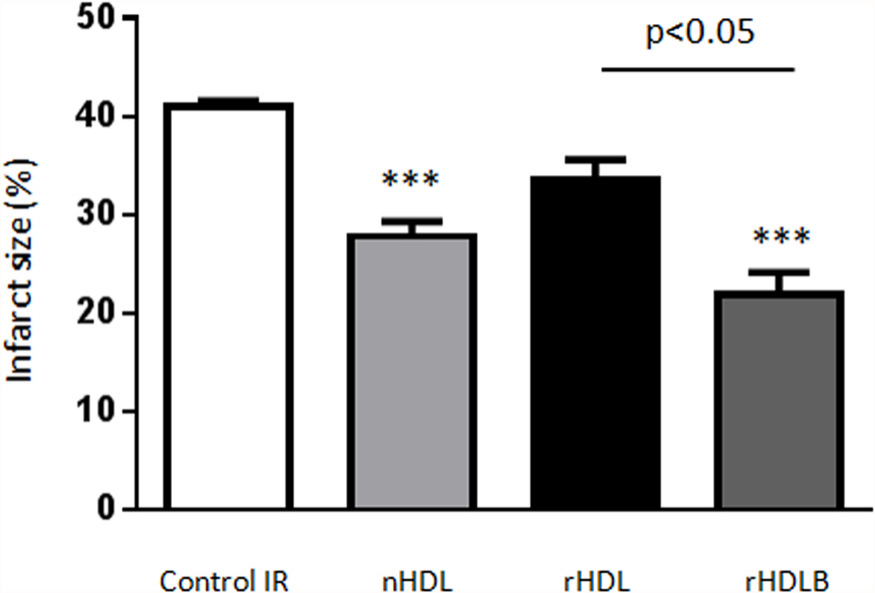
rHDL containing S1P protect against ischemia reperfusion injury *ex vivo*. Isolated hearts were submitted to global ischemia (35min) followed by reperfusion (45min). At the onset of reperfusion, hearts were treated or not (control) with native HDL (nHDL), rHDL (apoAI+POPC) or rHDLB (apoAI + POPC + S1P) during the first 7min. Infarct size is expressed as percentage of total heart area, (mean±SEM). ***p<0.001 vs control using one-way ANOVA combined with Tukey multiple comparisons post-hoc test, n≥4.

Analysis of the impact of rHDL on IRI in the isolated heart allowed us to eliminate potentially confounding, systemic, anti-inflammatory effects of HDL (for protocol details see Fig. 1A). Native HDL was used as a positive control. As shown in Fig. 3, native HDL reduced infarct size by 31.7%±3.9 (p<0.001). With respect to rHDL, treatment with basic rHDL (POPC+apoAI) did not significantly reduce infarct size (p = 0.056). Corresponding to the results observed *in vitro*, addition of S1P to basic rHDL resulted in a highly significant reduction in infarct size (Fig. 3; p<0.001), similar to that observed for native HDL.

**Fig 4:**
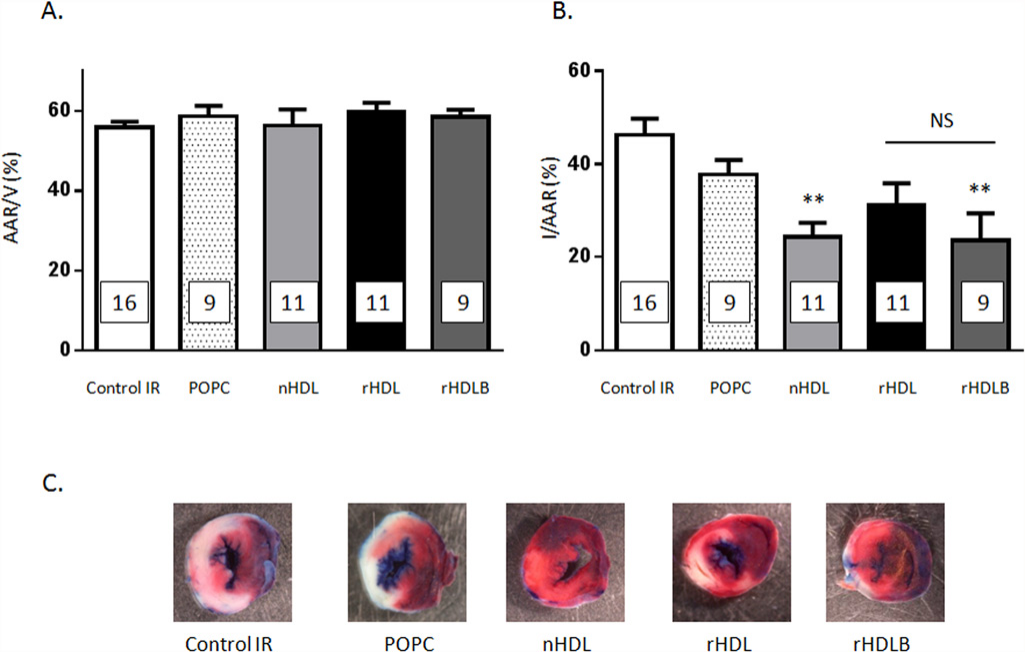
Post-ischemic treatment with HDL protects against ischemia reperfusion injury *in vivo*. Mice were submitted to LAD occlusion for 45min and hearts were reperfused for 24h. Mice were injected or not (control mice IR) with native HDL (nHDL), POPC, rHDL (apoA1+POPC) or rHDLB (apoAI + POPC + S1P) one minute before reperfusion. **A.** Quantification of area at risk (AAR) per ventricle surface. Data are mean±SEM (n = 9–16 per group). **B.** Quantification of infarct size (IS) expressed in % of AAR. Data are mean±SEM (n = 9–16 per group), **p<0.01, vs IR, using one-way ANOVA combined with Tukey multiple comparisons post-hoc test. **C.** Representative images of TTC stained middle heart sections of control or treated mice.

To assess the physiological relevance of our treatment protocol, the impact of HDL was examined in the *in vivo* model (for details see Fig. 1B). No significant difference between groups was observed with respect to the myocardial AAR (Fig. 4A). A single injection of native HDL immediately prior to reperfusion was sufficient to reduce this infarct size by 47.5%±6.8 (p<0.01) (Fig. 4B, 4C). Injection of the phospholipids (POPC) vector alone and basic rHDL (POPC+apoAI) (p = 0.52 and p = 0.053, respectively) did not have a significant impact on the infarct size (Fig. 4B, C). By contrast, addition of S1P to basic rHDL (rHDLB) significantly reduced infarct size to a degree equivalent to that obtained with native HDL (-49.2%±12.8, p<0.01; Fig. 4B, C).

The content of human apoAI in serum was measured after reperfusion. Human apoAI was still present in mouse serum 24h after reperfusion (see S2 Fig. and S3 Fig.). The content of S1P in HDL particles isolated from mouse serum was not significantly different between groups after 24h of reperfusion (see S4 Fig. and S5 Fig.).

### HDL do not modulate systemic inflammation

**Fig 5:**
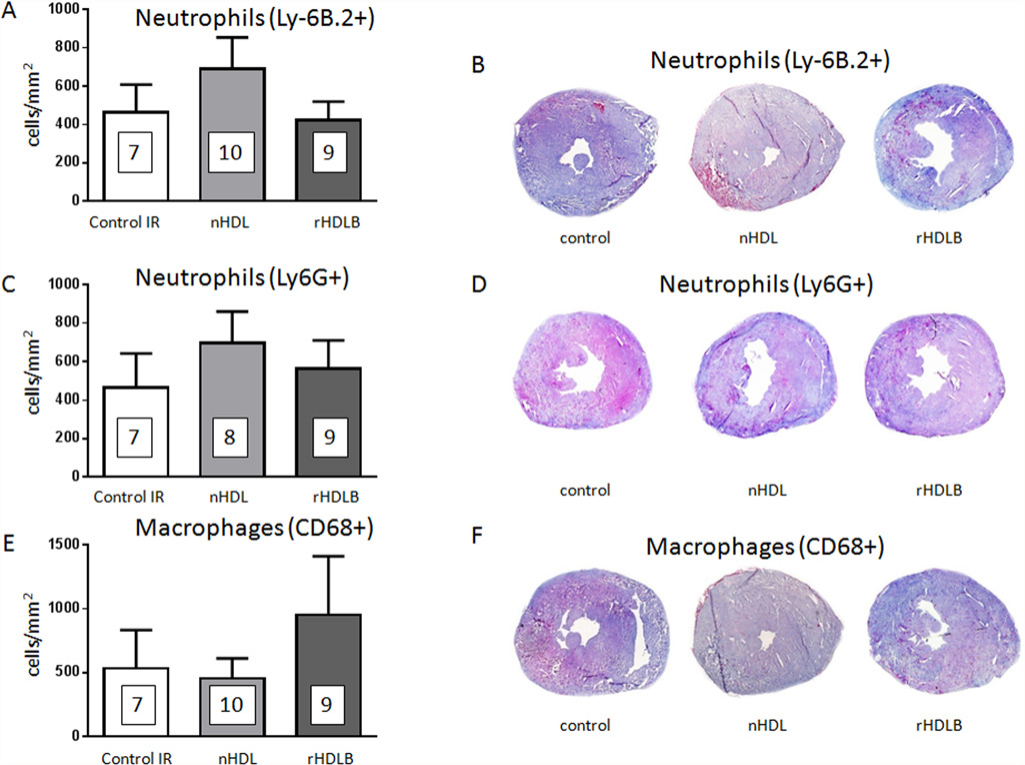
Post-ischemic treatment with HDL does not decrease leukocyte infiltration. Mice were submitted to LAD occlusion for 45min and hearts were reperfused for 24h. Mice were injected or not (control mice IR) with native HDL (nHDL), rHDLB (apoAI + POPC + S1P) one minute before reperfusion. **A.** Quantification of infiltrated neutrophils (Ly-6B.2^+^ cells) per area in frozen sections of infarcted hearts at 24h of reperfusion. **B.** Representative images of neutrophil (Ly-6B.2^+^ cells) infiltration. **C.** Quantification of infiltrated neutrophils (Ly6G+ cells) per area in frozen sections of infarcted hearts at 24h of reperfusion. **D.** Representative images of neutrophil (Ly6G+ cell) infiltration. **E.** Quantification of infiltrated macrophages (CD68^+^ cells) per area in frozen sections of infarcted hearts at 24h of reperfusion. **F.** Representative images of macrophage (CD68^+^ cells) infiltration. Data are expressed as scattered plots (mean±SEM, *n* = 7–10 per group). Results are expressed as percentages of stained area on total heart surface area. No significance difference between groups was found using unpaired-student t-test.

**Fig 6:**
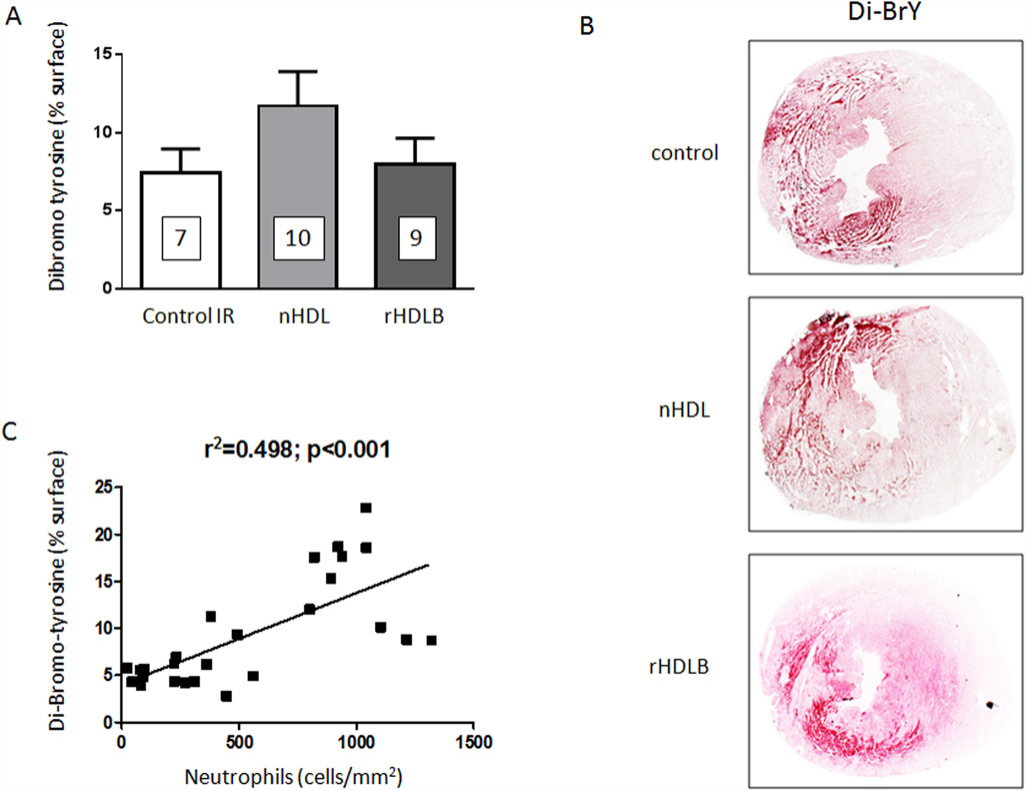
Post-ischemic treatment with HDL does not decrease oxidation. Mice were submitted to LAD occlusion for 45min and hearts were reperfused for 24h. Mice were injected or not (control mice IR) with native HDL (nHDL), reHDLB (apoAI + POPC + S1P) one minute before reperfusion. **A.** Quantification of Di-BrY content of frozen sections of infarcted hearts after 24h of reperfusion. **B.** Representative images of Di-BrY stained middle heart sections. **C.** Correlation between neutrophil content and Di-BrY staining after 24h of reperfusion in the same hearts.

HDL are known to modulate the inflammatory response. We investigated this possibility with the *in vivo* model. These experiments were limited to the most protective treatments: native HDL and rHDLB. We could not find evidence of a reduction in circulating levels of chemokines CCL2 or CXCL1 by treatment with native HDL or rHDLB, compared to controls (Table 1). Therefore, we extended our investigations to the inflammatory process within the cardiac tissue. As shown in Fig. 5, after 24h reperfusion neither native HDL nor rHDLB modulated neutrophil (Fig. 5A-5D) or macrophage (Fig. 5E, 5F) infiltration of the myocardial tissue compared to controls. Similarly, HDL treatment did not decrease myeloperoxidase-induced protein oxidation as measured using Di-BrY as a marker (Fig. 6A-6C). Moreover, histological analysis demonstrated that HDL treatment did not modulate myocardial lipid peroxidation (measured by 4-hydroxy-2-nonenal (4-HNE) staining) and superoxide (measured by dihydroethidium (DHE) staining) 24h after reperfusion (see S6 Fig.). Thus, one single injection of HDL at reperfusion did not significantly reduce the inflammatory response induced by ischemia.

**Table 1:** Circulating chemokines measured 24h after reperfusion.

chemokines	control	nHDL	p value vs control	rHDLB	p value vs control
**CCL2 (pg/ml)**	66.3 [46.1–229.1]	108.4 [57.5–223.7]	p = 0.3884	105.3 [38.4–125.8]	p = 0.776
**CXCl1 (pg/ml)**	412.0 [215.0–806.8]	395.2 [329.8–786.5]	p = 0.864	558.5 [281.9–840.0]	p = 0.607

Data are expressed as median (interquartile range [IQR]) of CCL2 or CXCL1 concentration (pg/ml). n = 7–9.

### HDL activate comparable intracellular signaling pathways *in vivo* and *in vitro*

**Fig 7:**
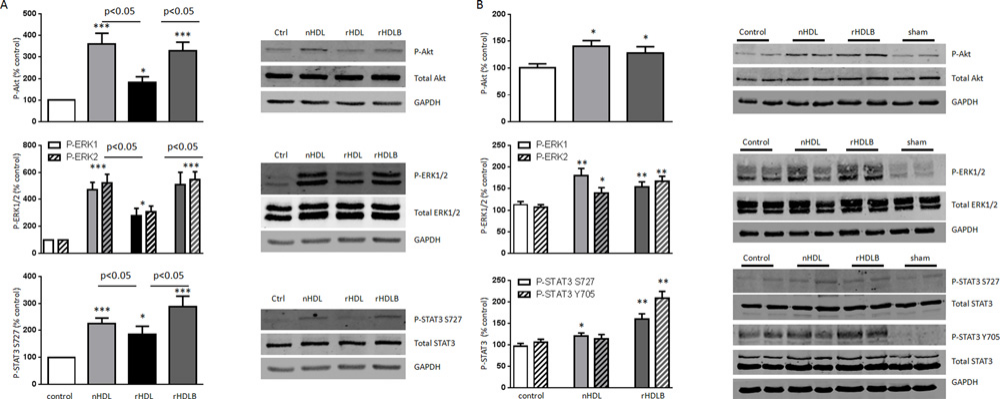
Activation of intracellular signaling pathways *in vitro* and *in vivo*. **A.** Western blot analysis was performed with proteins extracted from neonatal cardiomyocytes treated with nHDL, rHDL (apoAI + POPC) or rHDLB (apoAI + POPC+ S1P) for 5min (n = 7–11). Specific bands corresponding to phosphorylated Akt, ERK1/2 and STAT3 were quantified by densitometry, normalized using GAPDH expression and given as a percentage of the control. **B.** Western blot analysis of proteins extracted from hearts submitted to 45min of LAD occlusion followed by 5min of reperfusion. Native HDL (nHDL) or rHDLB were injected one minute before the reperfusion (n = 9). Data are mean±SEM. *p<0.05, **p<0.01, ***p<0.001 vs control, using unpaired-student t-test.

We analyzed survival pathways that were previous identified by our *in vitro* studies based on doxorubicin treatment of cardiomyocytes. The *in vitro* model revealed activation of Akt, ERK1/2 and STAT3 by native and rHDLB (Fig. 7A). Although basic rHDL induced the phosphorylation of these target proteins in cultured cardiomyocytes, treatment with HDL containing both apoAI and S1P (native HDL or rHDLB) enhanced these actions. These results were confirmed *in vivo* where Akt and ERK1/2 were activated by both native HDL and rHDLB (Fig. 7B). STAT3 was also phosphorylated, although it was more marked with rHDLB than with native HDL (Fig. 7B).

## Discussion

The study demonstrates that post-ischemic treatment with HDL, at the time of reperfusion, can strongly reduce the extent of myocardial IRI. Moreover, synthetic HDL is an appropriate substitute for native HDL, when S1P is added as it improves their protective capacity. Both native and synthetic HDL modulate the same cardiomyocyte signaling pathways, suggesting that they mediate cardioprotection by similar mechanisms. It implies that a direct action of HDL on cardiac cells is sufficient to limit IRI, irrespective of any impact on inflammatory factors.

The recent disappointing results of clinical trials that used HDL-cholesterol raising drugs to reduce vascular risk [14] have re-opened the debate about how to exploit the protective influence apparently mediated by HDL. One avenue, the use of rHDL as a therapeutic complex in man, is attracting attention. In experimental settings, administration of rHDL to patients was associated with improvements in plaque size, endothelial function and anti-inflammatory markers [15–19] as well as in glucose control and insulin secretion in diabetic patients [20]. In patients with acute coronary syndrome the use of rHDL leads to an improvement in the blood lipid profile [21]. Such studies have not investigated IRI and all have employed the basic rHDL formulation.

Our observations suggest that the therapeutic use of HDL could be extended to IRI in a clinically relevant acute setting. A limited number of older studies in animal models reported that rHDL could limit ischemic damage to the myocardium [2, 4]. These were, however, largely based on perfusion with rHDL prior to ischemia, which does not correspond to the real-life, clinical situation, and concerned essentially the *ex vivo*, whole heart model. Although treatment with rHDL at reperfusion has been showed to improve myocardial post-ischemic parameters, the improvement was reportedly much lesser extent than when rHDL were given before ischemia. Of note these studies did not evaluate the consequence of the treatment on the infarct size [2, 22].

Only two studies have reported on post-ischemic treatment with *in vivo* models. Kiya et al. [23] reported that rHDL limited left ventricular remodeling in a rat model. They employed, however, an experimental model with permanent ligation of a coronary artery and weekly HDL injections over 4 weeks. This model is clinically inapplicable for acute myocardial infarction. The second study in a rabbit model reported a beneficial effect on IRI of a synthetic HDL preparation infused for 60min, starting 5min before reperfusion [2, 5]. Our studies evaluated acute (only one injection) treatment with rHDL at reperfusion, which may explain some discrepancies. Nevertheless, our data extend the observations from these previous studies with respect of the improvement of the protective capacity of rHDL by addition of S1P that to our knowledge has never been investigated *in vivo*.

Although S1P receptor knockout models have provided powerful evidence for the importance of S1P in HDL-mediated cardioprotection, the role of the lipid is still debated. Our observations are interesting in this context. Results obtained in our experimental model showed that although rHDL of basic formulation had a positive effect, they were unable to significantly protect against IRI. Addition of S1P was necessary to reduce IRI in a significant manner. Interestingly, very recent data have shown that rHDL is a strong acceptor of S1P from erythrocytes [24]. The ability of HDL to acquire S1P from serum could explain the positive, albeit non-significant, impact of rHDL observed *in vivo*. Thus maximal clinical efficiency of rHDL would appear to require the presence of S1P. The data are consistent with S1P being the component of HDL that allows the latter to limit IRI [25]. Observations on the activation of signaling pathways concur with this conclusion. Indeed, both native and rHDL containing S1P (rHDLB) strongly activated ERK1/2, STAT3 and Akt, whereas basic rHDL was significantly less effective. In our preceding doxorubicin-based studies, we demonstrated the importance of S1P and the S1P2 receptor for activating ERK1/2 and STAT3 during HDL-mediated cardiomyocyte survival [26]. Our more recent studies further underline the role of STAT3 in HDL and S1P-induced cardioprotection [27, 28].

A previous study by Theilmeier et al. [3] attributed a major part of HDL protection to a nitric oxide dependent mechanism involving endothelial cells and observed reduced neutrophil and macrophage infiltration of cardiac tissue. In our study we could not find evidence of a modulated inflammatory response, or reduced leukocyte infiltration in the myocardium 24h after reperfusion. Although only one time point has been investigated, this is consistent with data from our 3 models (*in vitro*, *ex vivo* and *in vivo*), which indicate that a direct impact of HDL on the cardiomyocyte is sufficient to mediate cardioprotection.

In conclusion, the present study demonstrates that HDL afford strong protection against IRI when employed post-ischemia, where synthetic HDL can replicate the effects of native HDL if S1P is included in the formulation. A direct impact on the cardiac cell is suggested by our observations of 3 complementary models, with the same survival pathways being activated. The study supports the potential clinical use of rHDL for protecting against IRI, and underlines the importance of defining rHDL composition to achieve optimal efficacy of reperfusion strategies. The therapeutic use of rHDL in acute myocardial infarction merits further consideration.

## Supporting Information

S1 Methods This is the Supplementary Material and Methods. (DOCX)

S1 Fig S1P content measured in nHDL and rHDLB. The content of S1P in native and artificial rHDL was also evaluated and adjusted to apoAI content.
(TIF)

S2 Fig Concentration of human apoAI from mouse serum. Human apoAI in serum from control or HDL-, rHDL, rHDLB-injected mice (24h after reperfusion) was measured by turbinometry assay. *p<0.05 vs control mice, using one-way ANOVA combined with Tukey multiple comparisons post-hoc test (n = 4/group).
(TIF)

S3 Fig Representative Western blot of human apoAI in mouse serum. Circulating human apoAI was estimated in serum from non-treated (control IR) or nHDL-, rHDL-, rHDLB-injected mice 24h after reperfusion. Each line represents serum from individual mouse.
(TIF)

S4 Fig S1P content in HDL isolated from mouse serum. HDL from control or nHDL, rHDL, rHDLB-injected mouse serum was isolated by ultracentrifugation (n = 3–4). The concentration of S1P in HDL particle was analyzed by LC/MS—MS.
(TIF)

S5 Fig Representative SDS-PAGE of HDL isolated from mouse serum. HDL isolated from control or HDL-, rHDL, rHDLB-injected mice serum (1μl) were run in SDS-PAGE (acrylamide 12%). Total proteins were visualized using Commassie staining. This gel demonstrates the purity of HDL which content in S1P was analyzed by LC/MS—MS.
(TIF)

S6 Fig Post-ischemic treatment with HDL does not decrease oxidation. Mice were submitted to LAD occlusion for 45min and hearts were reperfused for 24h. Mice were injected or not (control mice IR) with native HDL (nHDL), rHDLB (apoAI + POPC + S1P) one minute before reperfusion. 4-HNE (A) and DHE (B) content of frozen sections of infarcted hearts at 24 h of reperfusion. Data are mean±SEM (n = 7–9 per group). B. Representative images of 4-HNE (C) and DHE (D) stained middle heart sections of vehicle, native HDL or rHDLB-treated mice at 24 h of reperfusion. No significance difference between groups was found using unpaired-student t-test.
(TIF)
