# Supplementary material for: Improving Reconstituted HDL Composition for Efficient Post-Ischemic Reduction of Ischemia Reperfusion Injury
Source: PLoS One. 2015 Mar 17;10(3):e0119664. doi: 10.1371/journal.pone.0119664 (PMC4362758; doi:10.1371/journal.pone.0119664)
Supplement: S1 Methods — (DOCX) [file pone.0119664.s001.docx]

**Supplementary materiel and methods**

**S1P measurement by liquid-chromatography/tandem mass spectrometry (LC/MS–MS)**

***Chemical and reagents***

Sphingosine-1-phosphate (S1P) was purchased from Tocris Bioscience (Bristol, United Kingdom) and S1P-d7 was purchased from Avanti Polar Lipids Inc. (Alabaster, AL, USA).

Stock solutions were prepared at 1.0 mg/mL (1 mg of substance in 1 mL DMSO/MeOH 1:4 + 4% HCl 30%). Working solutions were prepared by dilution in MeOH of the stock solutions to reach concentrations of interest ranging from 1 to 100 ng/mL. Stock and working solutions were stored at -80°C.

Ultra-pure water (for chromatography) was produced using a milli-Qplus 185 from Millipore (Volketswil, Switzerland). MeOH (HPLC grade) was obtained from Merck (Darmstadt, Germany) and formic acid (FA, MS grade) was obtained from Sigma-Aldrich (Buchs, Switzerland). HDL were supplied by the University Hospital of Geneva (Geneva, Switzerland).

***Preparation of biological samples.***

Isolated HDL were diluted 1:9 in MeOH containing 10 ng/mL internal standard (S1P-d7) prior injection into the LC-MS/MS system.

***Chromatographic and mass spectrometric conditions***

*Equipment*

Analyses were performed on LC-MS/MS system consisting of a 5500 QTrap ® triple quadrupole linear ion trap (QqQ_LIT_) mass spectrometer equipped with a TurboIon Spray™ interface (AB Sciex, Framingham, MA, USA) and an Ultimate 3000 Series (Thermo Fisher Scientific Inc., Whaltham, MA, USA) as LC system. Data acquisition and analysis were performed using Analyst^TM^ software (version 1.6.2; AB Sciex, Framingham, MA, USA).

*Chromatographic conditions*

Substances were separated using a Chromolith RP-18 analytical column (100 mm x 3 mm i.d.; Merck, Darmstadt, Germany). Mobile phase was constituted of a mixture of water (A) and MeOH (B) both with 0.1% FA at a flow rate of 0.6 mL/min. A gradient was used starting from 50 to 100% B in 1.5 min, maintained during 8 min, and re-equilibrated at 50% in 0.1 min during 1.4 min for an overall analysis time of 11 min. The autosampler was kept at 6°C. The measured retention times are 3.97 and 3.96, respectively for S1P and S1P-d7.

*MS detection*

The TurboIon Spray interface was operated in the negative ionization mode. The parameters of the source were used with the following settings using nitrogen as curtain and nebulizer gas: capillary voltage -4.0 kV, temperature 625°C, curtain gas 20 psi, collision gas -8, GS1 45 psi, and GS2 45 psi. For the MRM parameters, a dwell time of 50 ms, a declustering potential of -100 eV, and a collision energy of -40 eV were used for both S1P and S1P-d7. The precursor ions are 378.2 *m/z* and 385.2 *m/z* for S1P and S1P-d7, respectively. The same product ion of 78.8 *m/z was selected for both compounds.* In this mode, Q1 and Q3 resolutions were placed to give a 0.7 u at full width at half maximum (FWHM) for both precursor and product ions. The MS/MS experiments were based on collision-induced dissociation (CID) occurring in the collision cell (quadrupole 2), with nitrogen as collision gas set at 10.

***Validation procedure***

For each day of the validation procedure (n=3), 5 repetitions of the basal level were carried out to establish the response corresponding to the endogenous level of our pool of HDL. This measured basal concentration was then removed in all calculations for the validation procedure.

Based on the guidelines of the “Société Française des Sciences et des Techniques Pharmaceutiques” (SFSTP) [1], validation was carried out over three non-consecutive days (p=3). For each day, calibration samples (cal) were prepared in duplicate (n=2), at six concentrations levels (k=6; cal = 1, 2, 5, 10, 50, 100 ng/mL) for the determination of the response function of S1P for each day.

In the same way, quality control samples (QC) were prepared independently in quadruplicates (n=4), at four concentrations levels representing the calibration range (k=4; QC = 1, 5, 25, 100). Based on analysis of variance (ANOVA), specific criteria such as trueness, precision, accuracy, linearity and limit of quantification were determined. Matrix effect was also investigated [2].

***Validation***

A linear regression model gave us the most suitable response function, with a weighting factor of 1/x^2^.

*Trueness and precision*

Trueness represented the systematic error of the method and was calculated as the ratio between the absolute and the mean measured concentration. The trueness varied from 88 to 104%.

On the other hand, precision represents the random error of the method that is expressed by the repeatability (R_RSD_) and the intermediate precision (IP_RSD_). The intra- and inter-day precision was calculated by computing the relative standard deviation (RSD) at each concentration level of the QC. RSDs were found to be lower than 10%, for every tested concentration levels that is in agreement with the limits fixed by the validation guideline.

*Linearity*

The linearity was expressed as the capacity inside a concentration range to provide results directly proportional to the concentration contained within the sample. It was determined by fitting the back-calculated concentrations of the QCs as a function of the introduced concentrations and by applying the linear regression model based on the least square method. The correlation coefficient for S1P over the concentration range was of 0.99992.

*Accuracy profile and limit of quantification (LOQ)*

The sum of the trueness (bias) and the precision (standard deviation), called also accuracy, corresponds to the total error of the method showed in the Figure below.

The LOQ of S1P was set at 1 ng/mL, since the limit of quantification is defined to be the concentration showing a total error lower than 20%, as presented in Figure below.

Figure: Accuracy profile for the determination of S1P using a linear regression with a weighted factor of 1 / x^2^. The continuous line represents the trueness, the dashed lines are the upper and lower accuracy limits in relative values and the dotted lines are the upper and lower 20% tolerance limits.

*Matrix effect and recovery*

The matrix effect was determined by post-infusion of the deutered S1P-d7 after injection of the diluted HDL sample (n=6) on the LC-MS system. Neither suppression nor enhancement of the signal was observed at the specific S1P retention time.

**Human serum apoAI concentration measurement**

Human apoAI concentration was determined by immunoturbidimetric assay according to the manufacturer’s instructions (Analyticon Biotechnologies AG, catalog n°H0801).

**Oxidative stress determination (24h after reperfusion)**

Measurement of superoxide in myocardium submitted to ischemia reperfusion injury (IRI) was performed using the superoxide-sensitive dye dihydroethidium (DHE, Molecular Probes) [3]. Five frozen midventricular cardiac sections per animal (euthanized after 24h of reperfusion) were stained with 10µM DHE at 37°C for 30min in a light-protected and humidified chamber. In situ fluorescence was assessed using fluorescence microscopy and quantification performed with MetaMorph software v6.0.

The production of reactive oxygen species (ROS) was assessed by a second histological method based on the highly toxic product of lipid membrane peroxidation, 4- hydroxy-2-nonenal, using mouse anti-4- HNE monoclonal antibody at 1μg/ml (Oxis International Inc, Foster City, CA) [4]. Five frozen midventricular cardiac sections per animal (euthanized after 24 h of reperfusion) were stained and analyzed. Quantification was performed with MetaMorph software v6.0. Results were expressed as percentages of stained area on total heart surface area.

**References**

1. Hubert P, Nguyen-Huu JJ, Boulanger B, Chapuzet E, Cohen N et al. (2007) Harmonization of strategies for the validation of quantitative analytical procedures. A SFSTP proposal--part III. J Pharm Biomed Anal 45:82-96.
2. Thomas A, Hopfgartner G, Giroud C, Staub C (2009) Quantitative and qualitative profiling of endocannabinoids in human plasma using a triple quadrupole linear ion trap mass spectrometer with liquid chromatography. Rapid Commun Mass Spectrom 23:629-38.
3. Montecucco F, Lenglet S, Braunersreuther V,  Pelli G, Pellieux C et al. (2010) Single administration of the CXC chemokine-binding protein Evasin-3 during ischemia prevents myocardial reperfusion injury in mice. Arterioscler Thromb Vasc Biol 30:1371–1377.
4. Liu YH, Carretero OA, Cingolani OH, Liao TD, Sun Y et al. (2005) Role of inducible nitric oxide synthase in cardiac function and remodelling in mice with heart failure due to myocardial infarction. Am J Physiol Heart Circ Physiol 289:H2616–2623.
