# Supplementary figures and images for: Improving Reconstituted HDL Composition for Efficient Post-Ischemic Reduction of Ischemia Reperfusion Injury
Source: PLoS One. 2015 Mar 17;10(3):e0119664. doi: 10.1371/journal.pone.0119664 (PMC4362758; doi:10.1371/journal.pone.0119664)

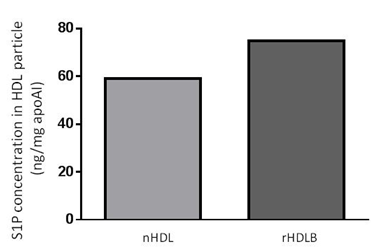

Supplement: S1 Fig — The content of S1P in native and artificial rHDL was also evaluated and adjusted to apoAI content. (TIF) [file pone.0119664.s002.tif]

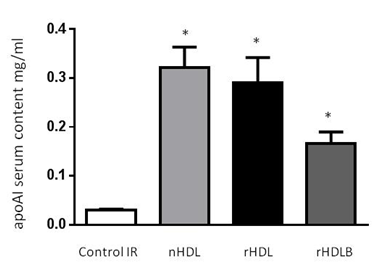

Supplement: S2 Fig — Human apoAI in serum from control or HDL-, rHDL, rHDLB-injected mice (24h after reperfusion) was measured by turbinometry assay. *p<0.05 vs control mice, using one-way ANOVA combined with Tukey multiple comparisons post-hoc test (n = 4/group). (TIF) [file pone.0119664.s003.tif]

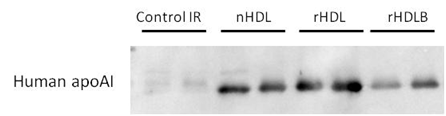

Supplement: S3 Fig — Circulating human apoAI was estimated in serum from non-treated (control IR) or nHDL-, rHDL-, rHDLB-injected mice 24h after reperfusion. Each line represents serum from individual mouse. (TIF) [file pone.0119664.s004.tif]

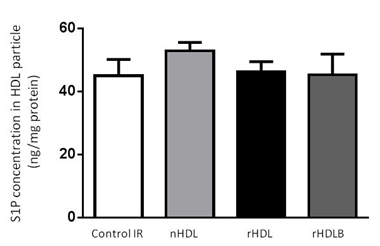

Supplement: S4 Fig — HDL from control or nHDL, rHDL, rHDLB-injected mouse serum was isolated by ultracentrifugation (n = 3–4). The concentration of S1P in HDL particle was analyzed by LC/MS—MS. (TIF) [file pone.0119664.s005.tif]

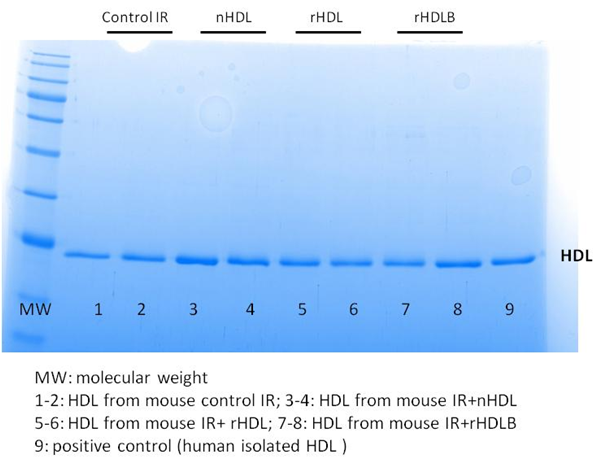

Supplement: S5 Fig — HDL isolated from control or HDL-, rHDL, rHDLB-injected mice serum (1μl) were run in SDS-PAGE (acrylamide 12%). Total proteins were visualized using Commassie staining. This gel demonstrates the purity of HDL which content in S1P was analyzed by LC/MS—MS. (TIF) [file pone.0119664.s006.tif]

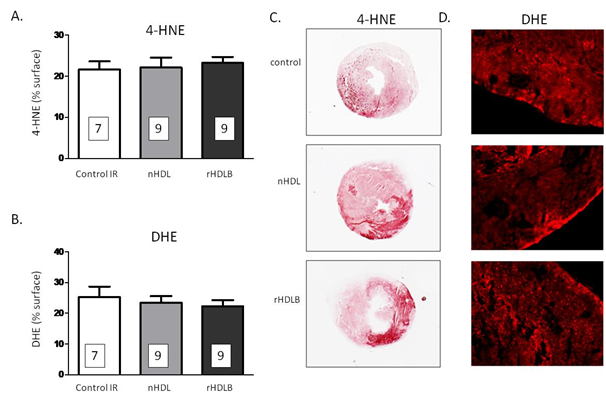

Supplement: S6 Fig — Mice were submitted to LAD occlusion for 45min and hearts were reperfused for 24h. Mice were injected or not (control mice IR) with native HDL (nHDL), rHDLB (apoAI + POPC + S1P) one minute before reperfusion. 4-HNE (A) and DHE (B) content of frozen sections of infarcted hearts at 24 h of reperfusion. Data are mean±SEM (n = 7–9 per group). B. Representative images of 4-HNE (C) and DHE (D) stained middle heart sections of vehicle, native HDL or rHDLB-treated mice at 24 h of reperfusion. No significance difference between groups was found using unpaired-student t-test. (TIF) [file pone.0119664.s007.tif]
